# Nuclear–Cytoplasmic Coevolution Analysis of RuBisCO in Synthesized *Cucumis* Allopolyploid

**DOI:** 10.3390/genes10110869

**Published:** 2019-10-30

**Authors:** Yufei Zhai, Xiaqing Yu, Zaobing Zhu, Panqiao Wang, Ya Meng, Qinzheng Zhao, Ji Li, Jinfeng Chen

**Affiliations:** State Key Laboratory of Crop Genetics and Germplasm Enhancement, College of Horticulture, Nanjing Agricultural University, Nanjing 210095, China

**Keywords:** allopolyploid, plastid–nuclear evolution, RuBisCO, *Cucumis*

## Abstract

Allopolyploids are often faced with the challenge of maintaining well-coordination between nuclear and cytoplasmic genes inherited from different species. The synthetic allotetraploid *Cucumis × hytivus* is a useful model to explore cytonuclear coevolution. In this study, the sequences and expression of cytonuclear enzyme complex RuBisCO as well as its content and activity in *C. × hytivus* were compared to its parents to explore plastid–nuclear coevolution. The plastome-coded *rbcL* gene sequence was confirmed to be stable maternal inheritance, and parental copy of nuclear *rbcS* genes were both preserved in *C. × hytivus.* Thus, the maternal plastid may interact with the biparentally inherited *rbcS* alleles. The expression of the *rbcS* gene of C-homoeologs (paternal) was significantly higher than that of H-homoeologs (maternal) in *C. × hytivus* (HHCC). Protein interaction prediction analysis showed that the rbcL protein has stronger binding affinity to the paternal copy of rbcS protein than that of maternal copy in *C. × hytivus*, which might explain the transcriptional bias of the *rbcS* homoeologs. Moreover, both the activity and content of RuBisCO in *C. × hytivus* showed mid-parent heterosis. In summary, our results indicate a paternal transcriptional bias of the *rbcS* genes in *C. × hytivus*, and we found new nuclear–cytoplasmic combination may be one of the reasons for allopolyploids heterosis.

## 1. Introduction

Allopolyploidization, including interspecific hybridization and whole-genome duplication, has played a key role in the evolution of plant species [1,2,3]. Previous studies have indicated that almost all ancestors of seed plants have experienced at least one round of genome doubling [4,5]. Allopolyploidization often results in complex changes at different aspects, such as genomic changes including homoeologous exchanges and loss of genes [6,7,8], nonadditive gene expression [9,10], and changes in epigenetic modifications [5,11]. During allopolyploidization, not merely two different nuclear genomes combine, but also different sources of chloroplast and mitochondrial genomes interact in the same cell, in view of the complex interactions between the nuclear and organelle genomes [12]. Cytonuclear interaction had been considered as an effective mean for eukaryotes to produce diverse phenotypes, and to improve the competitiveness of survival and reproduction [13,14]. Allopolyploidization disrupts nuclear–cytoplasmic interactions, including changes in gene copy number and stoichiometry [2]. For most angiosperms, organelles (plastid and mitochondrial) are maternally inherited [15]; to date, only very low-frequent biparental or paternal transmission of plastid DNA has been reported in few plant species [16,17,18]. Therefore, in the case of the universal uniparental maternal inheritance of plastid genome in plants, allopolyploids are expected to be more closely matched to maternal homoeologs of plastid targeted genes than to paternal homoeologs [19]. Ribulose-1, 5-bisphosphate carboxylase/oxygenase (RuBisCO), which is localized to chloroplast stroma, is composed of small subunits encoded by the nucleus and large subunits encoded by plastid, and is an ideal system to study the evolution process of plastid–nuclear interactions. Gong et al. [20] found a consistent transcriptional bias of the maternal *rbcS* homoeologs in four natural allopolyploids, *Arabidopsis*, *Arachis*, *Brassica*, and *Nicotiana*. Nevertheless, study in synthesized rice allotetraploids has shown there was no consistent pattern of biased expression of maternal-like *rbcS* homoeologs [21]. A recent study revealed subgenome dominance towards the A subgenome (paternal progenitor) of nuclear genes involved in plastid protein complexes in resynthesized and natural *Brassica napus* [22]. Hence, cytonuclear coevolution appears to be a complicated but underexplored aspect of allopolyploidization, the genetic and evolutionary forces that allopolyploidization imposes upon cytonuclear interaction still need more comprehensive and systematic study to be well understood.

Synthesized allopolyploids can be very useful models to study cytonuclear interaction since it presents a new nuclear–cytoplasmic combination and can be tested on a known genetic background. An interspecific cross was successfully made in *Cucumis* between wild *Cucumis* species, *C. hystrix* Chakr. (HH, 2n = 2x = 24) and cultivated cucumber, *C. sativus* L. ‘BeijingJietou’ (CC, 2n = 2x = 14) [23]. The chromosome numbers of *C. hystrix × C. sativus* F1 interspecific hybrid (HC, 2n = 19) were then doubled through somaclonal variation using embryo culture technique, and a synthesized allotetraploid species, *C. × hytivus* Chen and Kirkbride (HHCC, 2n = 4x = 38), was obtained [24]. This allotetraploid can self-pollinate to produce viable seeds, providing a unique system to reveal the complicated processes during allopolyploid evolution and speciation.

The genome of cucumber has three different genetic patterns; the chloroplast, mitochondrial, and nuclear genomes are maternally, paternally, and biparentally transmitted, respectively [25,26]. For *C. × hytivus*, Shen et al. [27] concluded that mitochondrial DNA was paternally inherited, while chloroplast DNA was maternally inherited between *Cucumis* species. Thus, the newly synthetic allotetraploid *C. × hytivus* combines two nuclear genomes but inherits only one set of progenitor organellar genomes, respectively, providing a unique model system for analyzing early cytonuclear evolution in allopolyploidization. In this study, the plastid-nuclear coevolution of RuBisCO encoding genes, nuclear *rbcS* and plastid *rbcL* genes, as well as RuBisCO content and activity in allotetraploid *C. × hytivus* and its diploid parents, were characterized. We aimed to explore the RuBisCO in the early stages of allopolyploidization to gain insight into the pace of cytonuclear coevolution of *C. × hytivus*.

## 2. Materials and Methods

### 2.1. Plant Materials, DNA, and RNA Extraction

Three species of *Cucumis* were used for this study: the cultivated cucumber *C. sativus* ‘BeijingJietou’ (2n = 14, genome CC), the self-cross plants (S14) of the synthesized new allotetraploid species *C. × hytivus* (2n = 38, genome HHCC), and the wild species *C. hystrix* (2n = 24, genome HH). The two diploid plants used in this experiment are the same inbred lines used in the original interspecific cross that generated the interspecific F1. The initial allotetraploid was obtained by chromosome doubling through somaclonal variation using embryo culture technique. The allotetraploid *C. × hytivus* used in this study was the fourteenth generation (S14) of self-cross plants.

The extraction of total genomic DNA was done using a modified cetyltrimethylammonium bromide (CTAB) method [28]. Total RNA was extracted using Trizol (Invitrogen, Carlsbad, CA, USA) and digested with DNase I for 30 min at 25 °C to remove DNA. Then, 2 µg of total RNA was used to synthesize complementary DNA (cDNA) using a cDNA Synthesis Kit (Fermentas, York, UK).

### 2.2. Gene Cloning and Quantitative Real-Time PCR Analysis

Primers used to amplify the full-length genomic and CDS sequence of *rbcL* and *rbcS* genes in three *Cucumis* species are listed in Table S1. Polymerase chain reactions (PCRs) were performed as described in Gong et al. [29], and the resulting clones were sequenced (Tsingke, Beijing, China). When amplifying the *rbcS* genes in allotetraploid *C. × hytivus*, three parallel independent PCRs for each primer sample were performed, the PCR products were cloned into the pMD-19T vector (Takara, Shiga, Japan) and the resulting all clones were sequenced. To exclude the possibility of PCR recombination and sequencing errors, each sequence was tested at least twice, and only the *rbcS* copies that sequenced at least 25% supportive clones in each independent PCR experiment were accepted as true copies. The sequences in this study have been uploaded to the GenBank database (accession numbers MK948862–MK948868; https://www.ncbi.nlm.nih.gov/genbank/). Species-specific single-nucleotide polymorphisms (SNPs) were deduced from alignments of the full-length sequence of genomic *rbcS* orthologs in parental diploids and that of homoeologs in allopolyploid. 

The expression of *rbcL* genes was analyzed by quantitative real-time PCR (qRT-PCR) using the SYBR Premix Ex TaqTM Kit (Takara) as described by Li et al. [30]. The *β-Actin* gene and *F-box* gene were used to quantify the relative transcript levels. The relative expression levels of *rbcL* genes were calculated following the 2^−ΔΔCT^ method [31] with the normalization of data to the geometric average of the internal control genes [32]. The gene-specific primers used for expression analysis were listed in Table S1.

### 2.3. Quantification and Comparison of rbcS Aallelic and Homeologous Expression Based on RNA-Sequencing

Three biological replicates of next-generation RNA-sequencing (RNA-seq) data of the allotetraploid and diploid parents were downloaded from the SRA database at NCBI (https://www.ncbi.nlm.nih.gov/sra) with accession number SRP155470 (Table S2). The samples of RNA-seq data were the leaves of seedlings of three species under normal growth conditions (14/10 h day/night, 28 ºC/20 ºC day/night, light intensity 500 μmol/m^2^s^1^ and air humidity controlled around 70%) at the same developmental stage. Raw data (raw reads) were filtered to produce clean data with high quality. Transcriptome assembly was accomplished based on the ‘left.fq’ and ‘right.fq’ using Trinity [33] fragments per kilobase of transcript per million fragments (FPKM) was used for the evaluation of expression of the *rbcS* transcripts in three species of *C. hystrix* (HH), *C. × hytivus* (HHCC) and *C. sativus* (CC).

The H- and C-*rbcS* homoeolog expression levels in allotetraploid *C. × hytivus* were analyzed according to the method of Gong et al. [29]. The raw reads of each replicate from *C. × hytivus* RNA-seq data were mapped onto the *rbcS* cDNA sequence using the Burrows–Wheeler alignment tool [34]. The GATK2 software was used to call SNPs [35]. Raw vcf files were filtered using the GATK standard filter method, and only SNPs with distance > 5 bp were retained. The read number based on specific nucleotide sites of homoeo-SNPs detected in the allotetraploid *C. × hytivus* was searched. In this way, we can calculate the readings number covering the H-genome homoeo-SNPs and the C-genome homoeo-SNPs, which represented the H- and C-*rbcS* homoeolog expression levels in *C. × hytivus*, respectively.

### 2.4. The Prediction of RuBisCO Protein–Protein Complex Binding Affinity

The predicted dissociation constant (Kd) and binding free energy (ΔG) of RuBisCO protein–protein complex were obtained from PPA-Pred2 [36] based on the protein sequences of rbcL and rbcS in *C. sativus*, *C. × hytivus*, and *C. hystrix*.

### 2.5. Measurement of RuBisCO Activity and Content

RuBisCO activity and content were measured by enzyme-linked immunosorbent assay (ELISA) using Plant RuBisCO ELISA Kit (SenBeiJia Biotechnology Co., Ltd. Nanjing, China) of the SBJ-P1004-48T and SBJ-Pl031-48T, respectively. About 1 g leaves were ground to a fine powder, and 9 mL extraction solution was added. The sample was then obtained by centrifugation at 4 ºC, 8000 g for 10 min, and the supernatant was taken for the next steps according to the manufacturer’s instructions. The kits use the purified plant RuBisCO antibody to coat the microtiter plate, to prepare solid-phase antibody, then add RuBisCO to the microwell of the coated monoclonal antibody, followed by HRP labeling. The antibody binds to form an antibody-antigen-enzyme antibody complex, which is thoroughly washed and then added to the substrate TMB for color development. Finally, the optical delnsity (OD) value of absorbance was measured with a microplate reader, and the activity and content of RuBisCO in the sample were calculated from the corresponding standard curves. All the samples were performed in three biological replicates. The mid-parent heterosis (MPH) was calculated using the following formula: MPH = (allotetraploid value − mid-parental value)/mid-parental value in %, where allotetraploid value is the average value of *C. × hytivus*, and the mid-parental value is the average value of the two parents (*C. hystrix* and *C. sativus*).

### 2.6. Statistical Analysis

The qRT-PCR results were expressed as the mean ± standard deviation (SE) of three experimental replicates. The values were subjected to Duncan’s multiple range testing with the SPSS 16.0 (SPSS Inc., Chicago, IL, USA). The binomial test was used to assess the bias in *rbcS* homeologous expression in *C. × hytivus*, in which the expression of the parental *rbcS* homeologous gene was set to the success and failure of the ‘binom.test’ function in R programming language (https://www.r-project.org/) (with the hypothesized probability of success being *p* = 0.5), respectively.

## 3. Results

### 3.1. Sequence Variation of RuBisCO Encoding Genes in Cucumis Allopolyploid

Plastome-encoded *rbcL* gene and nuclear-encoded *rbcS* gene in *C. × hytivus* and parental diploids were cloned and sequenced. There was only one copy of the *rbcL* ortholog and *rbcS* ortholog gene in diploid parents, *C. hystrix* (HH) and *C. sativus* (CC). 

**Figure 1:**
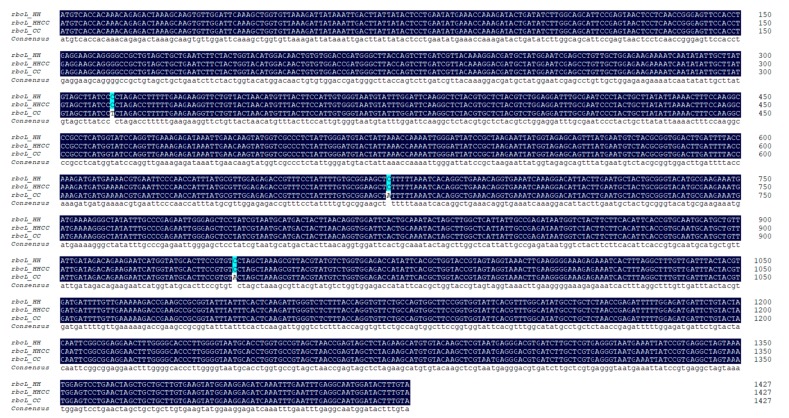
Sequence alignment of allopolyploid *Cucumis × hytivus* (HHCC) RuBisCO coding gene *rbcL* with those from the progenitor diploid (*C. hystrix*, HH and *C. sativus*, CC) *rbcL* genes. The cloned *rbcL* gene in sampled allotetraploid *C. × hytivus* is identical to the *rbcL* gene in its maternal subspecies *Cucumis hystrix* (HH).

**Figure 2:**
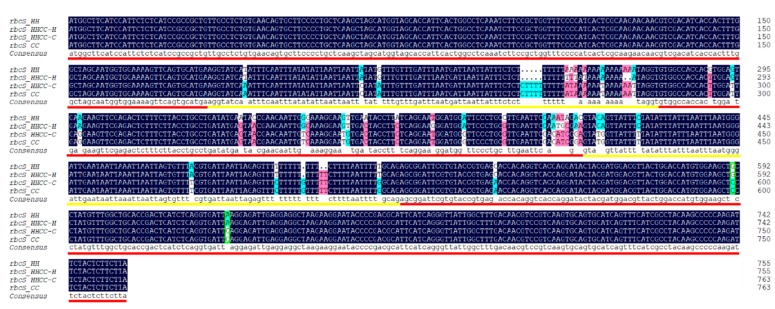
Sequence alignment of allopolyploid *C. × hytivus* (HHCC) RuBisCO coding gene *rbcS* with those from the progenitor diploid (HH and CC) *rbcS* genes. The red and yellow lines represent the three exons and two introns, respectively. Homoeo-SNPs (homoeo-single nucleotide polymorphisms) at positions 599 (where HH orthologs had a G, but CC orthologs had a C) and 636 (where a HH orthologs had an A, but CC orthologs had a C) are annotated in green boxes.

Three SNPs were detected between the parental *rbcL* orthologs (Figure 1), among the three SNP, one is synonymous, and the other two are non-synonymous (Figure S1). Parental *rbcS* genes consist of three exons and two introns, and 33 SNPs and three indels were detected between the parental *rbcS* orthologs, of which 8 SNPs (8/33) and all indels (3/3) occurred within the two introns (Figure 2). Corresponding rbcS amino acid sequence alignment shows 15 amino acid differences (Figure S2).

The *rbcL* gene in *C. × hytivus* was confirmed to show maternal inheritance (Figure 1). Sequence alignments of the *rbcS* gene showed there were two types of *rbcS* genes in *C. × hytivus* (HHCC), predicted CC allele was identical with CC *rbcS*, whereas the HH allele has some mutations compared to the HH *rbcS* gene, including eight free non-directional mutations and 12 mutations that tend to CC *rbcS* (Figure 2). Sequence alignment combined with homoeo-SNPs detected in *C. × hytivus* identified two species-specific SNPs (Figure 2): one of which is located at position 599 in the *rbcS* genomic sequence (corresponding to position 393 in the cDNA alignment), wherein the HH orthologs have a G but CC orthologs have a C, and the other is located at position 636 in the *rbcS* genomic sequence (corresponding to position 430 in the cDNA alignment), wherein HH orthologs have an A, but CC orthologs have a C. These SNPs distinguished between paralogs from the H and C genomes in allotetraploid, therefore, these two species-specific SNPs can be used to estimate homoeolog expression in *C. × hytivus* (HHCC).

### 3.2. Expression of Maternal Inheritance of the rbcL Gene and Duplicated rbcS Genes in Allotetraploid C. × hytivus

**Figure 3:**
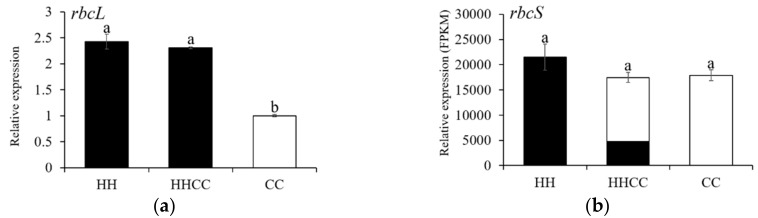
(**a**) *rbcL* homoeolog expression and (**b**) *rbcS* homoeolog expression in *C. hystrix* (HH), *C. × hytivus* (HHCC), and *Cucumis* *sativus* (CC). The expression of *rbcL* and *rbcS* genes was analyzed in three biological replicates by qRT-PCR and RNA-seq, respectively. H subgenome (black, female parent), C subgenome (white, male parent). Data represents the mean ± standard deviation (SE) (*n* = 3). The different lower-case letters above the bars show the means are significantly different according to Duncan’s multiple range test at 5% level.

In order to understand how RuBisCO encoded genes perform following genome merger and doubling, qRT-PCR was performed to analyze the expression of *rbcL* genes in three species of *C. hystrix* (HH), *C. ×hytivus* (HHCC), and *C. sativus* (CC). The *rbcL* transcriptional level in CC was significantly lower than that in HH and HHCC, and there was no significant difference between HH and HHCC (Figure 3). The expression level of the *rbcS* gene was analyzed using the method of fragments per kilobase of transcript per million fragments (FPKM) based on the databases of genomic RNAseq, and no significant difference in the expression of the *rbcS* gene was detected between the two parental diploids HH and CC, suggesting that there is an equal amount of transcription of the *rbcS* genes relative to the total transcriptome in H- and C-genome progenitors (Figure 3).

The parental origin of each *rbcS* and homoeolog in the allotetraploid *C. × hytivus* can be distinguished by using homoeo-SNPs. The aforementioned two species-specific SNPs were used to estimate homoeolog expression to assess whether there was a biased expression of *rbcS* genes in *C. × hytivus*. In all three replicates of *C. × hytivus* RNA-seq libraries, the observed total expression of C-homoeologs (indicated by C subtotal in Table 1) was significantly higher than that of H-homoeologs (indicated by H subtotal in Table 1) in allopolyploid *C. × hytivus* (HHCC). Therefore, *rbcS* expression has significant genomic bias in the direction of its paternal genome donor (Figure 3 and Table 1). 

### 3.3. The Prediction of the RuBisCO Protein–Protein Complex Binding Affinity

In order to find out the possible reasons for paternally-biased *rbcS* gene expression in *C. × hytivus.*

PPA-Pred2 was used to predict dissociation constants and binding free energy (ΔG) of RuBisCO complex formed by rbcL and rbcS proteins in *C. sativus*, *C. × hytivus*, and *C. hystrix*, respectively. The results showed that diploid parents have higher binding efficiency than tetraploid, CC diploid’s rbcL and rbcS has higher binding free energy than that of HH diploid. Within the allopolyploid *C. × hytivus*, rbcS C-homolog has a lower dissociation constant and higher binding free energy for rbcL relative to H homolog (Table 2). The values observed suggest that rbcS C-homoeologs have a higher affinity to rbcL than that of H-homoeologs. The result indicates that the paternal copy of rbcS protein had a stronger interaction with rbcL protein, which possibly explained the transcriptional bias of the paternal-like *rbcS* homoeologs. 

### 3.4. The Influence of Allopolyploidization on RuBisCO Content and Activity

To investigate how allopolyploidization affects the function of RuBisCO, we measured and compared the RuBisCO content and activity of *C. × hytivus* with its diploid parents. When normalized for fresh weight, both RuBisCO content and activity in leaves of *C. × hytivus* showed positive mid-parent heterosis (MPH, mean that heterotic traits outperform to the average of its two parents) (6.9% and 14.32%, respectively) (Table 3). The result indicates that *C. × hytivus* show hybrid vigor of RuBisCO. 

## 4. Discussion

Allopolyploid speciation often faces the challenges of stoichiometric disruption due to the combination of two divergent nuclear genomes and only one parental set of organelle genomes [21]. Changes in one genomic compartment may influence other genomes evolution in a cell [37]. The RuBisCO is a cytonuclear enzyme complex, making it an ideal system to study plastid–nuclear coordination. It is often used as a window to explore the cytonuclear evolutionary features of allopolyploidization. In the case of allopolyploid *C. × hytivus*, the maternal plastid interacts with the biparentally inherited nuclear rbc*S* alleles. The predicted CC allele in *C. ×hytivus* (HHCC) was identical with CC diploid r*bcS* gene, whereas the HH allele has some mutations compared to HH diploid *rbcS* gene, including some mutations without direction and some mutations that tend to CC *rbcS*, presumably due to being affected by subgenomic dominant or subsequent nucleo-cytoplasmic interactions. The observed total expression of C-homoeologs (paternal) was significantly higher than that of H-homoeologs (maternal) in allopolyploid *C. × hytivus* (HHCC) in its RNA-seq libraries. This result indicates a significant genomic bias in *rbcS* expression in the direction of its paternal genome donor in *C. × hytivus*. The ratio of rbcS: rbcL expression is much higher in CC than in HH or HHCC, there is such a possibility that rbcS’s relatively high expression in CC diploid appears to carry over to the CC homoeolog in the allotetraploid, and cis regulation may play a role in regulating gene expression. Beyond that, the protein–protein interaction prediction indicates that the paternal copy of the rbcS proteins had a stronger interaction with rbcL protein, which might explain the transcriptional bias of the paternal-like *rbcS* homoeologs. We point out that the heterologous expression of chloroplast-associated nuclear genes in allopolyploids may be related to chloroplast function. Although the expression bias of homoeologous genes in allopolyploids has been reported to be related to nuclear genome stability, studies on the relationship between organelles and nuclear stability have been ignored [38]. We hope this research will be a stepping stone to explore the relationship between chloroplasts and mitochondria with the different parental genome behaviors. 

Previous studies about cytonuclear coevolution in allopolyploids reported transcriptional bias of the maternal *rbcS* homoeologs were detected in five natural allopolyploids, including *Arabidopsis*, *Arachis*, *Brassica*, *Gossypium*, and *Nicotiana* [20,29]. However, our research and several recent studies of allopolyploids [21,22,39] do not support the hypothesis of preferential expression of maternal transcripts. In addition to the influence of nucleus-organelle interactions, the expression bias of *rbcS* in *C. × hytivus* could also be the weak selection pressure from the limited non-synonymous substation between diverged H- and C-genome rbcL or insufficient evolution time allowed for such initial synthesized HHCC to achieve the ultimate cytonuclear coevolution. The expression of individual genes varies greatly [40], and it does not represent the global genomic bias in transcription, but its related studies can supplement the mechanism of subgenome transcriptome asymmetry. Subgenome expression of both synthetic and natural allopolyploids was conditioned by parental legacy and modified by transcriptome shock. However, natural allopolyploids also experience cumulative effects during the evolutionary process under natural and/or human selection [41]. Different regulatory modules may cause inconsistent expression bias in allopolyploids. We conclude that cytonuclear evolution after genome doubling is a necessary and complicated coevolving process, the direction of evolution could be related to different types of species and evolutionary time.

Differential gene expression can play a role in coordinating cytonuclear interactions in allopolyploids [19]. Allopolyploids may have the advantage of integrating more genetic resources through genome reorganization and flexible gene expression [42]. Chen [43] systematically characterized the roles of nonadditive gene expression, epigenetic regulation, and small RNAs in hybrid vigor. And in this study, both the activity and content of RuBisCO in *C. × hytivus* showed mid-parent heterosis (MPH) (Table 3), indicating *C. × hytivus* showed hybrid vigor of RuBisCO. We propose that new nuclear–cytoplasmic combination may be one of the reasons for heterosis in allopolyploid. Another possibility of the departure from mid-parent expectations in allopolyploid may be the outcome of separate inheritance of the nuclear vs. cytoplasmic genomes. It may be possible to verify this theory through a synthetic reciprocal combination. A better understanding of the regulatory mechanisms for heterosis in allopolyploid will help us efficiently select the better combinations of parents to produce the best performing hybrids and polyploids. Further study of the accommodation in other cytonuclear coencoded complexes (including mitochondria and chloroplast related proteins), and comprehensive analysis over generations may provide valuable information on the mechanism of heterosis and the pace of cytonuclear co-evolution.

## 5. Conclusions

Here, synthesized *Cucumis* allopolyploid was used to explore the cytonuclear interaction of RuBisCO. The nuclear *rbcS* gene responded to nuclear–cytoplasmic interactions in the new genetic systems, specifically showed transcriptional paternal bias expression, which supports that maternally-biased gene expression of the cytonuclear enzyme complexes does not always occur in allopolyploid. This pattern of transcriptional bias is highly likely to be related to the physical and chemical properties of protein, in other words, the maternal plastid-encoded rbcL can have priority for the biparental nuclear-encoded rbcS with higher affinity. Our results point out the contribution of protein interaction affinity in the evolution of nuclear genes involved in plastid protein complexes, and we suggest that new nuclear–cytoplasmic combination may be one of the reasons for heterosis in allopolyploid. It is necessary to carry out extensive research on other cytonuclear enzyme complexes in allopolyploids.

## Figures and Tables

**Table 1:** rbcS homoeolog expression in *Cucumis* allopolyploid *C. × hytivus.*

	Homoeolog	SNP:393	SNP:430
HHCC-1	H Subtotal	687	687
	C Subtotal	959	805
	Total	1646	1445
HHCC-2	H Subtotal	458	359
	C Subtotal	603	439
	Total	1061	794
HHCC-3	H Subtotal	440	369
	C Subtotal	660	558
	Total	1100	927

**Table 2:** Prediction of the binding affinity of protein –protein complex of rbcL and rbcS.

rbcL	rbcS	Kd (Dissociation Constant)	ΔG (Binding Free Energy)
rbcL HHCC	rbcS HHCC-H	1.51·10^-07^ M	−9.30 kcal/mol
	rbcS HHCC-C	6.87·10^−08^ M	−9.77 kcal/mol
rbcL HH	rbcS HH	9.30·10^−09^ M	−10.95 kcal/mol
rbcL CC	rbcS CC	8.19·10^−09^ M	−11.03 kcal/mol

HHCC-H: HH allele in HHCC, HHCC-C: CC allele in HHCC.

**Table 3:** RuBisCO content and activity in *C. hystrix* (HH), *C. × hytivus* (HHCC), and *C. sativus* (CC).

	HH	HHCC	CC	MPH (%)
RuBisCO content (ng/g FW)	1.41 ± 0.03	1.70 ± 0.03	1.77 ± 0.02	6.90%
RuBisCO activity (μmol/min/g FW)	7.24 ± 0.17	6.86 ± 0.10	4.76 ± 0.14	14.32%

FW:Fresh weight; MPH: Mid-parent heterosis. Values represent the mean ± SE (*n* = 3), the experiment was performed in three biological replicates.

## Supplementary Materials

The following are available online at https://www.mdpi.com/2073-4425/10/11/869/s1, Figure S1: Alignment of translated CDS regions of allopolyploid *C. × hytivus* (HHCC) with those from the progenitor diploid (HH and CC) *rbcL* genes, Figure S2: Alignment of translated CDS regions of allopolyploid *C. × hytivus* (HHCC) with those from the progenitor diploid (HH and CC) *rbcS* genes, Table S1: The sequence of primers employed in this study, Table S2: The RNA-seq data used in this study.
