# Supplementary material for: Nuclear–Cytoplasmic Coevolution Analysis of RuBisCO in Synthesized Cucumis Allopolyploid
Source: Genes (Basel). 2019 Oct 30;10(11):869. doi: 10.3390/genes10110869 (PMC6895982; doi:10.3390/genes10110869)
Supplement: Supplementary file 1 [file genes-10-00869-s001.pdf]

Supplementary Material:

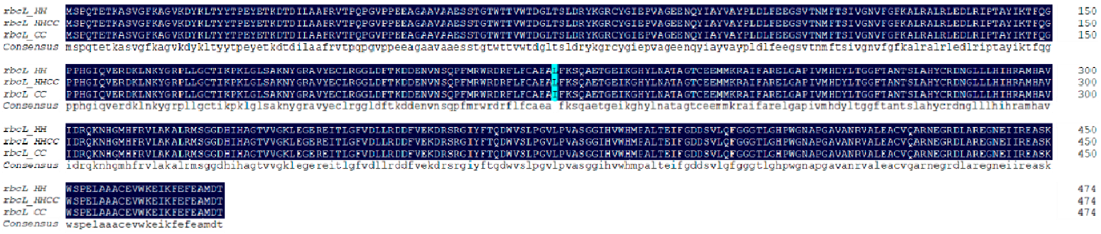

Figure S1. Alignment of translated CDS regions of allopolyploid *C. ×hytivus* (HHCC) with those from the progenitor diploid (HH and CC) *rbcL* genes.

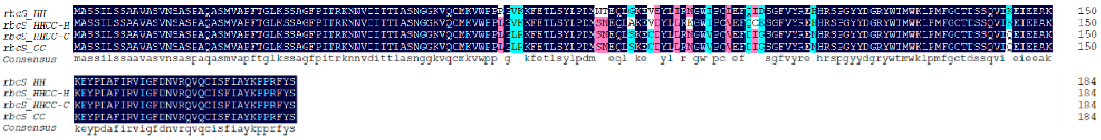

Figure S2. Alignment of translated CDS regions of allopolyploid *C. ×hytivus* (HHCC) with those from the progenitor diploid (HH and CC) *rbcS* genes.

Table S1. The sequence of primers employed in this study.

Primers	Sequence 5′→3′
**qRT-PCR analysis primers**	
q-CP-rbcL-F	CAGAGACTAAAGCAAGTGTTGG
q-CP-rbcL-R	GGTTGAGGAGTTACTCGGAAT
CsActin-F	ATTGTTCTCAGTGGTGGTTCTAC
CsActin-R	CCTTTGAGATCCACATCTGCT
F-box-F	GGTTCATCTGGTGGTCTT
F-box-R	CTTTAAACGAACGGTCAGTCC
***rbcL* and *rbcS* cloning primers**	
RbcS-F	GGCATAAATGGCTTCATCC
RbcS-R	GCAGATGGAACTTAAGAAGAGTAG
RbcL-F	TGTAGGGAGGGACTTATGTCAC
RbcL-R	TTGTATTCGGCTCAATCCTT

F forward primer, R reverse primer.

Table S2. The RNA-seq data used in this study.

SRA accession number	species	library name (three biological replicates)
SRP155470	*Cucumis hystrix*	Chys 12 1
		Chys 12 2
		Chys 12 3
	Cucumis ×hytivus	Chyt 10 1
		Chyt 10 2
		Chyt 10 3
	Cucumis sativus	Csat 8 1
		Csat 8 2
		Csat 8 3
